# Consolidation of the Two Associations

**Published:** 1897-05

**Authors:** B. H. Catching

**Affiliations:** Atlanta, Ga.


### CONSOLIDATION OF THE TWO ASSOCIATIONS.

BY DR. B. H. CATCHING, ATLANTA, GA.

Editorials, correspondence, and observation have, for some time, indicated a necessary change in the national dental organization of the United States. It has not required the closest observation to see that neither the American nor the Southern Dental Association is measuring up to the standard required by the advanced position of the dental profession. They have served a good purpose, and are now ready to give way to an organization which shall unite the profession as a whole, and render results commensurate with the calling of which it should be the representative.

There should be no obstacle thrown in the way of such an organization. While some may have feelings of strong attachment to the existing associations, yet they should lay sentiment aside for professional advancement. The men who have been in the forefront of professional life and progress will almost to a man join in this move.

From a sectional stand-point there may have once existed a reason for the two separate organizations. That reason no longer has force in the minds of those who think above sectionalism. The spirit dominating the mind of the sectionalist is not the spirit of progression, but of retrogression. There are enough men in the dental profession whose minds are sufficiently cultivated and enlarged to form an organization which shall represent advanced thought and progress of the age.

This is not the time to set forth plans for such an association, but it is not out of place to commence the plan as proposed by Dr. Harlan, who, as president of the American Dental Association in 1891, made the suggestion of dividing the country into quarter sections,—namely, northeastern, southeastern, northwestern, and southwestern, each division to have an organization to be known by the name of its respective territory; each division to meet anually, working under the same rules. From the four divisions or sections organize a national association to meet quadrennially at a given point, at which place would be a museum, library, and apparatus for research.

This seems to be an admirable suggestion, one not only feasible but practicable. This country is too large to be well represented annually in one organization. Distances are too great. As it stands, one-half the country is virtually denied the privilege of a national gathering. The quarterly division of the country, with an organization in each, would give that location which has been denied the benefit of organization, save State associations, vast advantages. Neither the American nor the Southern Dental Association can hold successful meetings west of the Mississippi River. Neither can the American have a large meeting if held in the South, especially if the time is in the summer months.

Every section of the country would be benefited by such a division of territory. Each section would vie with the other in good work. There would be a stimulus given to dental meetings that is badly needed; such a stimulus would bring forth work deserving a high calling, and lift association work out of the ruts in which it has been running for many years. Everything can be said in favor of this plan and nothing can be said against it.

